# Evaluating clinical stop‐smoking services globally: towards a minimum data set

**DOI:** 10.1111/add.14072

**Published:** 2017-11-26

**Authors:** Andrew L. Skinner, Robert West, Martin Raw, Emma Anderson, Marcus R. Munafò

**Affiliations:** ^1^ MRC Integrative Epidemiology Unit (IEU) at the University of Bristol Bristol UK; ^2^ United Kingdom Centre for Tobacco Control Studies and School of Experimental Psychology University of Bristol Bristol UK; ^3^ United Kingdom Centre for Tobacco Control Studies and Department of Epidemiology and Public Health University College London London UK; ^4^ United Kingdom Centre for Tobacco Control Studies and Division of Epidemiology and Public Health University of Nottingham Nottingham UK; ^5^ School of Social and Community Medicine University of Bristol Bristol UK

**Keywords:** Cessation, data, global, minimum, set, smoking

## Abstract

**Background and aims:**

Behavioural and pharmacological support for smoking cessation improves the chances of success and represents a highly cost‐effective way of preventing chronic disease and premature death. There is a large number of clinical stop‐smoking services throughout the world. These could be connected into a global network to provide data to assess what treatment components are most effective, for what populations and in what settings. To enable this, a minimum data set (MDS) is required to standardize the data captured from smoking cessation services globally.

**Methods:**

We describe some of the key steps involved in developing a global MDS for smoking cessation services and methodologies to be considered for their implementation, including approaches for reaching consensus on data items to include in a MDS and for its robust validation. We use informal approximations of these methods to produce an example global MDS for smoking cessation. Our aim with this is to stimulate further discussion around the development of a global MDS for smoking cessation services.

**Results:**

Our example MDS comprises three sections. The first is a set of data items characterizing treatments offered by a service. The second is a small core set of data items describing clients’ characteristics, engagement with the service and outcomes. The third is an extended set of client data items to be captured in addition to the core data items wherever resources permit.

**Conclusions:**

There would be benefit in establishing a minimum data set (MDS) to standardize data captured for smoking cessation services globally. Once implemented, a formal MDS could provide a basis for meaningful evaluations of different smoking cessation treatments in different populations in a variety of settings across many countries.

## Introduction

Despite the prevalence of tobacco use decreasing in many countries, there are still more than 1 billion tobacco users world‐wide 1, and this number is not falling. This is partly because of increasing prevalence in other countries and partly because of population growth. Some of these users may be able to stop when they choose, but the success rates of unaided quit attempts 2 and findings from clinical trials and population studies 3 indicate that most would benefit from support in their attempt to stop smoking. Article 14 of the World Health Organization Framework Convention on Tobacco Control (FCTC) requires Parties to take effective measures to promote tobacco use cessation and adequate treatment for tobacco dependence. Guidelines for the implementation of Article 14 were adopted in November 2010 and outline what is needed to enable Parties to meet their obligations under that Article 4. Current evidence, however, indicates that only a small minority of countries, and very few low‐ and middle‐income countries, have the infrastructure and systems elements in place to be able to meet these obligations 5, 6.

Studies in some high‐income countries suggest that specific medicines and types of behavioural support can improve smokers’ chances of long‐term success at stopping 7. However, in order to apply this work globally we ideally need to know more about their effectiveness throughout the world. More broadly, there is considerable room for improvement in stop‐smoking support in all settings. We need to build incrementally on our understanding of how combinations of behaviour change techniques, delivered in what way, to what kinds of smoker and in what settings provide the optimal outcomes 7.

In the future, the challenge for tobacco use cessation will be to improve upon the strategies currently available and, more importantly, to identify support options that are clearly effective, cost‐effective and affordable in different populations and different regions. Randomized controlled trials (RCTs) can only take us so far with this, and suffer from the well‐established limitations of generalizability, practicability, cost and time‐scale 8. A programme of research is required, complementing the existing evidence base from RCTs, to provide vital information about what approaches work most effectively throughout a broad range of settings. This programme should consist of quasi‐experimental and epidemiological studies that examine variations in smoking cessation outcomes among different cohorts of smokers using different methods of quitting, adjusting for as many confounding factors as possible 9. This kind of approach has already paid dividends in the United Kingdom, where the combination of a common national standard for outcome assessment 10 and the centralized collection of mandatory smoking cessation service (performance data by the Health and Social Care Information Centre, now NHS Digital) has enabled confirmation of findings from RCTs concerning the relative effectiveness of different forms of medication, and identification of specific treatments with improved success rates 11.

Our vision is to extend the success of the approach used in the United Kingdom (where there is still scope for capturing and using more data about treatments and clients for further analyses and optimization of services), and to deploy it globally. By connecting clinical smoking cessation services from many countries to form a global network of service providers, it will be possible to share information about the performance of different treatments in different scenarios within a common frame of reference, and to assess and identify optimal treatment strategies for different populations in different settings on a global scale. A key step in connecting providers to form a global network will be establishing a minimum data set (MDS) that should be captured by all clinical smoking cessation services in the network. The use of MDSs for the standardized capture of data is widespread in many aspects of health care, from the treatment and management of individual clinical conditions 12 and specific health‐care procedures 13 to entire disciplines 14. MDSs have also been used for evaluating smoking cessation services at a national level in a number of countries 15, 16.

Our aim here is to highlight the need for a global MDS for smoking cessation services, and to outline the steps towards developing a data set that can be used by all services, in all territories, of all countries. We describe a number of methods that could be used to produce this global MDS. We also provide an example of what a global MDS for smoking cessation could look like. Our intention is not to propose this as a candidate for a global MDS, as more thorough applications of the methodologies described, robust validations and detailed considerations of issues such as cross‐cultural differences are required to achieve this. Rather, we hope that it will stimulate further discussion concerning the need for a global MDS, and how best to achieve this.

An important aspect of the MDS is that it will be designed to be completed by the people that operate the smoking cessation services. This sets it apart from surveys gathering data for global initiatives for evaluating tobacco use and control, such as the World Health Organization (WHO)'s Framework Convention on Tobacco Control 17 and Global Adult Tobacco Survey 18, which are designed to be completed by governments and policy setters, and aim typically to capture larger volumes of more complex data. There will, however, be important lessons to be learnt from experiences with these surveys, including a range of cross‐cultural issues 19.

## Methods

A variety of methods can be used to develop a MDS 20. Most begin with some form of bottom‐up identification of candidate data items. This can include interviews with expert and stakeholder groups 21 and reviews of existing information systems and data sets 14. This is followed by a second stage, in which there is an iterative process of consolidation which aims to achieve consensus on optimal items, resolve conflicts and harmonize language, descriptions and formats, etc. One formal technique used increasingly for this second stage is Delphi 22; for examples see 14, 23. Finally, proposed data sets need to be validated. Again, a number of approaches are possible, most of which involve review by additional groups of experts and stakeholders, with methods such as the Nominal Group Technique 24 used to reach consensus on the applicability of items in data sets. The development of a formal global MDS for smoking cessation will need to follow these lines, using robust methods to identify and validate data sets, with particular attention given to testing of cross‐cultural aspects.

In order to stimulate discussions around the need for such MDS and the form this might take, we used informal approximations of these methods to produce an example global MDS for smoking cessation services. We used existing smoking cessation data sets from the UK's Stop Smoking Services and MDSs from other countries to identify candidate data items. To minimize repetition of treatment‐specific data, we defined separate data sets that describe the treatments offered by services, and the client's engagement with the various treatments offered by a service.

For the treatment data, we took as our starting point a list of behaviour change techniques found in UK Stop Smoking Services (SSS) 25. From this we selected only those treatment features found to be associated with improved quit outcomes [either self‐reported or carbon monoxide (CO)‐verified]. For client data, we began with the data items listed in the latest version (version 9) of the UK's National Centre for Smoking Cessation and Training's Stop Smoking Service Client Record Form 26. This template form has all the data items required for the routine performance assessment conducted of UK smoking cessation services. The form has some limitations; for example, there is possible ambiguity in how to complete items such as: ‘are you being treated for physical or mental health problems’, and it is possible that if an individual has a problem but is not being treated that the problem will not be recorded. However, any approach to asking these questions that attempts to balance accuracy of data with sensitivity concerning what constitutes a problem, in the absence of a full diagnostic procedure, will encounter some issue of this kind. The data required to describe a client's engagement with smoking cessation services can be extensive, and regional differences in resources (e.g. computer equipment, network infrastructure, measurement devices) will mean that the extent of data that can be captured by services will vary. We therefore propose, rather than a single client data set, a core set of data that could be captured in every smoking cessation service setting, and an extended, richer set of data items that could be captured wherever resources make this possible. Our sample MDS therefore comprises the treatment data, the core client data and the extended client data.

Data sets were then refined with reference to existing smoking cessation MDSs, namely the Scottish smoking cessation MDS 15 and the MDS for evaluation of North American smoking quitlines 16. Through an informal, iterative process of review and modification all authors, who have extensive experience of international smoking cessation services and epidemiological studies of tobacco use, then refined the example data sets further, and reached consensus on their contents. When developing a formal MDS a formal process for iteratively reaching consensus, such as Delphi 22, should be considered for this stage.

To provide some level of cross‐cultural validation, we then sought feedback on the example data sets from colleagues in the International Cooperation Centre for the Framework Convention on Tobacco Control in Uruguay and the Centre for Tobacco Studies in Syria. Feedback was provided by one representative from each organization. In both cases the feedback was positive, with agreement that the choice of data items for each data set was broadly appropriate for those services in those settings. Issues raised included the limited availability of CO measurement in some regions, labels used for some data items (e.g. ‘gender’ would be more acceptable than ‘sex’), and how best to capture some data (e.g. ‘age’ would be preferable to ‘date of birth’). The example data sets were updated accordingly. Again, the process used to reach agreement for the sample MDS was informal, but formal approaches such as Nominal Group Technique should be considered when developing the formal MDS.

In broader cross‐cultural terms, it is important to note that not all countries have sufficient infrastructure to support dedicated clinical smoking cessation services. In those that do not, there is often more of a focus on delivery of opportunistic brief advice supporting smoking cessation through alternative existing mechanisms, such as tuberculosis clinics 27, 28. These brief advice approaches vary considerably from the services offered by smoking cessation clinics, largely because they are not designed to follow‐up and track client quit attempts. The data describing brief advice approaches will therefore vary significantly from data describing clinical smoking cessation service, and we suggest that defining minimum data sets for brief advice approaches might best be handled as a separate (but linked) activity.

## Results

Example treatment data that should be captured once for each treatment a service offers are shown in Table 1. Example core client data that should be captured in every clinical smoking cessation service setting are shown in Table 2. Example extended client data that should be captured where resources permit are shown in Table 3.

**Table 1:** Treatment information (one record per type of treatment on offer).

Item label	Definition	Response options
Name of service	Name of current service	Free text
Name of subservice	Name of component of current service	Free text
Service setting	Type of environment in which service is delivered	One from: community/ psychiatric/ hospital/ pharmacy/ dental/ general practice/maternity/ children's centre/educational/ prison/military base/ other
Treatment mode	The mode of the treatment provided	One from: closed group/ couple/family/telephone/text message/support/ one‐to‐one support/open (rolling) group/ drop‐in clinic
Average session length	Length of sessions in minutes, taking account of the fact that the first session is often longer	Length in minutes
Number of sessions	Total number of sessions	Number of sessions
Frequency of sessions	Number of sessions per week	Number of sessions/week
Duration of behavioural support	Number of weeks post‐quit date that support is provided	Number of weeks
**Content of behavioural support**		
Strengthen ex‐smoker identity	Advise on the importance of ‘not a puff no matter what’ and starting to consider smoking as ‘not an option’	Yes/No
Elicit client views	Check client's understanding and ensure that s/he has an opportunity to ask questions and express concerns	Yes/No
Measure CO	At the first session this provides an indication to the smoker and the practitioner of the degree of toxin exposure from smoking; after the quit date provides confirmation of smoking abstinence	Yes/No
Explain the purpose of CO monitoring	Provides motivation to the client not to smoke after the quit date and rewards the client for not smoking	Yes/No
Give options for additional and later support	Offer additional phone or text message support	Yes/No
Provide rewards contingent upon stopping smoking successfully	Be fulsome in praise for not smoking at each post‐quit visit	Yes/No
Advise on changing routine	Discuss with the client, and get agreement to, ways of avoiding specific smoking triggers	Yes/No
Facilitate relapse prevention and coping	Discuss with client specific ways of dealing with cravings when they arise without smoking	Yes/No
Advise on stop smoking medicine	Try to ensure that the smoker agrees to use the most effective stop‐smoking medication available in the locality (ideally either varenicline or NRT patch plus a faster acting product)	Yes/No
Ask about experiences of stop smoking medication that the smoker is using	Check that the client is using the stop‐smoking medication properly and address any concerns about adverse effects	Yes/No
Advise on conserving mental resources	Advise on how to ensure that client gets enough sleep and minimizes exposure to stress	Yes/No
Advise on/facilitate use of social support	Discuss with client ways in which s/he can get the support of friends or family	Yes/No
Summarize information/confirm client decisions	Provide a summary of the key points of each session and up to three things to keep in mind between it and the next session Confirm client's decisions and commitments made during the session	Yes/No
Provide reassurance	Address client's concerns and provide reassurance that adverse or worrying experiences are normal and will subside	Yes/No
Boost motivation and self‐efficacy	Express belief in the client's ability to succeed, and help the client to believe that s/he will succeed	Yes/No
Provide information on withdrawal symptoms	Ensure that the client knows what to expect in terms of the nature, severity and duration of withdrawal symptoms	Yes/No

CO = carbon monoxide; NRT = nicotine replacement therapy.

**Table 2:** Core client data.

Item label	Definition	Response Options
Age	Client's age in years	Integer
Gender	Client's gender	Male | Female
Usual daily cigarette consumption	Number of cigarettes smoked usually smoked each day	Integer
Date of initial contact	When the client first visits the service.	Day/Month/Year
Pharmacological support used	All of the pharmacological supports planned to be used by client	Any from (dummy coded) Varenicline | Bupropion | Cytisine | Nortriptyline | NRT | Nicotine vapourising device | Other
NRT support used	All of the types of NRT planned to be used by client	Any from (dummy coded) None |Patch | Gum | Lozenge | Nasal Spray | Mouth Spray | Oral Strips | Inhalator | Microtab | Other
4 weeks: self‐report no puff in past 2 weeks	No smoking at all in the past 2 weeks at 4‐week follow up	Yes | No | Lost to follow up
12 weeks: self‐report no puff in past 10 weeks	No smoking at all in the past 4 weeks at the 12‐week follow up	Yes | No | Lost to follow up

**Table 3:** Extended client data.

Item label	Definition	Response options
**Adviser information**		
Adviser ID	The service adviser's unique identification number, assigned by the service's national coordinating body	Integer assigned by the system
Venue ID	A unique code for the service site, assigned by the service's national coordinating body	Integer assigned by the system
**Client information**		
Pre‐quit CO reading	Measure of carbon monoxide (p.p.m.) in expelled air before quit attempt	Integer
Pregnant	Is the client pregnant?	Yes/No
Breastfeeding	Is the client breastfeeding?	Yes/No
Education level	Years of formal education	One from: none/primary/secondary/tertiary/Bachelors/Masters/Doctoral
Occupation	Category of client's occupation	One from: managerial/professional/intermediate/routine and manual/home carer/retired/never worked, long‐term unemployed/unable to work (sick/disabled)/prisoner
Currently treated for physical health problem?	Is the client currently being treated for any physical health problems?	Yes/No
Currently treated for mental health problems?	Is the client currently being treated for any mental health problems?	Yes/No
Currently treated for drug or alcohol problem?	Is the client currently being treated for any alcohol or drug‐related problems?	Yes/No
Current medications	Any medication the client is currently taking	Select from fixed list (with typing completion) plus specified other
Partner smoking status	Does the client's partner smoke?	Yes/no/not applicable
Do others smoke in your house?	Do people other than your partner smoke in your house?	Yes/no
Time to first cigarette of the day	How many minutes until client has first cigarette of the day?	One from: within 5 minutes/6–30 minutes/31–60 minutes/> 60 minutes
Willing to quit?	Client's willingness to set a target quit date	Yes/no
Self‐efficacy	Client's confidence in their ability to quit	Five‐point scale from 1 (very low) to 5 (very high)
Agreed quit date	Date the client plans to stop smoking completely, with support from service	Day/month/year
How much time currently spent with urges to smoke (prior to quitting)?	How much time does the client currently spend with urges to smoke?	One from: none of the time/a little of the time/some of the time/a lot of the time/almost all the time
How strong are the urges (prior to quitting)?	How strong are the client's urges to smoke?	One from: no urges/slight/moderate/strong/extremely strong
Weeks since most recent quit attempt	How many weeks since the client's most recent attempt to quit smoking?	Integer
How long recent quit attempt lasted	How many weeks did the client's most recent attempt to quit last?	Integer
Past use of stop smoking medicines	Any medicines the client has used in previous attempts to quit	Any from: varenicline/bupropion/cytisine/nortriptyline/NRT/nicotine vapourizing device/other
If multiple licensed pharmacological supports used	Are these used at same time or consecutively?	One from: used at same time/used consecutively
How client heard about the service	How the client heard about the service	One from: GP/other health professional/friend/relative/advertising/pharmacy/other
**Outcome data**		
Pre‐quit CO reading	Measure of carbon monoxide (p.p.m.) in expelled air before quit attempt	Integer
4 weeks: CO reading	Measure of carbon monoxide (p.p.m.) in expelled air at 4 week visit	Integer
4 weeks: CO verified 4‐week quitter	Quit status at 4 weeks (< 10 p.p.m.)	Yes/No/not available
12 weeks: CO reading	Measure of carbon monoxide (p.p.m.) in expelled air at 12 week visit	Integer
12 weeks: CO‐verified 12‐week quitter	Quit status at 12 weeks (<10 p.p.m.)	Yes/No/not available

CO = carbon monoxide; p.p.m. = parts per million; NRT = nicotine replacement therapy; GP = general practitioner.

## Discussion

While individual tobacco cessation services using locally defined measures and methods may be effective to some degree, harmonizing the data captured throughout services will make it possible to assess the effectiveness of different treatments in different regions, to identify optimal sets of approaches for supporting smoking cessation in a variety of different settings and to commission more effective and cost‐effective measures that can reach more smokers and increase quit rates. This approach has proved successful in the United Kingdom through standardization of outcome assessments and the use of common data structures in the systems supporting services, although there is still scope for capturing additional data for further analyses and service optimization. Our vision is to extend this approach and deploy it internationally. To enable this vision, a minimum data set is required for the standardized capture of data from smoking cessation services globally. In this study, we take the first steps towards this by highlighting the need for a global MDS for smoking cessation services, and outlining some of the key steps and methodologies that should be considered in developing this.

To stimulate further discussion concerning the need and development of a global MDS for smoking cessation, we used informal approximations of some of the methods discussed here to produce an example MDS. To be clear, our aim with this is to facilitate further detailed discussion about the development of a global MDS; we are not suggesting this be considered as a proposal for a formal global MDS. The informal approaches used to develop this sample MDS lack the rigour of the formal approaches we have suggested. Development of a proposal for a global MDS will require the full application of these formal techniques for identifying candidate data sets and achieving consensus among experts and stakeholders, robust techniques for evaluation of proposed data sets and detailed assessments and testing of cross‐cultural factors. Cross‐cultural aspects will be important, and should consider issues such as: variations in resources for, and acceptability of, capturing data items between countries and between territories with varying resources within countries; issues around data integrity and ownership; and regional differences in tobacco use. These issues should be given particular attention when considering how the MDS will be used in low‐ and middle‐income countries, where smoking rates are rising, and the use of an MDS to facilitate service optimization could potentially have the greatest impact.

We invite comments and suggestions on any aspect of the development of a global MDS for smoking cessation, including which methods will be optimal for development and validation of the MDS, and specific cross‐cultural aspects that should be considered. We propose that these comments and suggestions be collated by the newly formed International Centre for Tobacco Cessation (ICTC), a centre set up to offer countries technical assistance in improving their tobacco cessation support systems, of which one of us (M.R.) is director (all communications on this topic should be addressed to martin@martinraw.com). Beyond gathering this feedback, the next stages will be to develop a formal MDS, promote use of the MDS in smoking cessation services globally, maintain the MDS moving forward (so that, for example, details of new harm reduction products can be added as they come to market) and collate and analyse data captured using the MDS from cessation services. We propose that these tasks should also be handled by the ICTC, although we welcome suggestions for alternative approaches.

## Declaration of interests

Robert West has received travel funds and hospitality from, and undertaken research and consultancy for pharmaceutical companies that manufacture or research products aimed at helping smokers to stop. These products include nicotine replacement therapies, Champix (varenicline) and Zyban (bupropion). This has led to payments to him personally and to his institution. He undertakes lectures and training in smoking cessation methods which have led to payments to him personally and to his institution. He has received research grants from medical charities and government departments. He is an unpaid advisor to the UK's National Centre for Smoking cessation and Training.
